# Bridging the Resolution Gap in Structural Modeling of 3D Genome Organization

**DOI:** 10.1371/journal.pcbi.1002125

**Published:** 2011-07-14

**Authors:** Marc A. Marti-Renom, Leonid A. Mirny

**Affiliations:** 1 Structural Genomics Laboratory, Bioinformatics and Genomics Department, Centro de Investigación Príncipe Felipe, Valencia, Spain; 2 Harvard-MIT Division of Health Sciences and Technology, and Department of Physics, Massachusetts Institute of Technology, Cambridge, Massachusetts, United States of America; University of California San Diego, United States of America

## Abstract

Over the last decade, and especially after the advent of fluorescent *in situ* hybridization imaging and chromosome conformation capture methods, the availability of experimental data on genome three-dimensional organization has dramatically increased. We now have access to unprecedented details of how genomes organize within the interphase nucleus. Development of new computational approaches to leverage this data has already resulted in the first three-dimensional structures of genomic domains and genomes. Such approaches expand our knowledge of the chromatin folding principles, which has been classically studied using polymer physics and molecular simulations. Our outlook describes computational approaches for integrating experimental data with polymer physics, thereby bridging the resolution gap for structural determination of genomes and genomic domains.

**This is an “Editors' Outlook” article for** ***PLoS Computational Biology***

Recent experimental and computational advances are resulting in an increasingly accurate and detailed characterization of how genomes are organized in the three-dimensional (3D) space of the nucleus (Figure 1) [1]. At the lowest level of chromatin organization, naked DNA is packed into nucleosomes, which forms the so-called chromatin fiber composed of DNA and proteins. However, this initial packing, which reduces the length of the DNA by about seven times, is not sufficient to explain the higher-order folding of chromosomes during interphase and metaphase. It is now accepted that chromosomes and genes are non-randomly and dynamically positioned in the cell nucleus during the interphase, which challenges the classical representation of genomes as linear static sequences. Moreover, compartmentalization, chromatin organization, and spatial location of genes are associated with gene expression and the functional status of the cell. Despite the importance of 3D genomic architecture, we have a limited understanding of the molecular mechanisms that determine the higher-order organization of genomes and its relation to function. Computational biology plays an important role in the plethora of new technologies aimed at addressing this knowledge gap [2]. Indeed, Thomas Cremer, a pioneer in studying nuclear organization using light microscopy, recently highlighted the importance of computational science in complementing and leveraging experimental observations of genome organization [2]. Therefore, computational approaches to integrate experimental observations with chromatin physics are needed to determine the architecture (3D) and dynamics (4D) of genomes.

**Figure 1:**
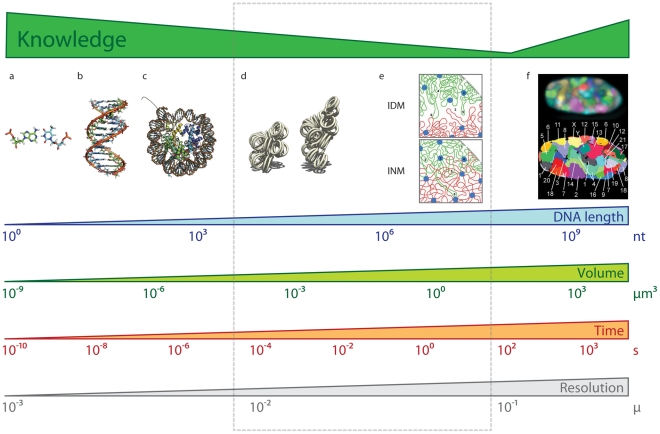
Bridging the resolution gap. DNA and chromatin have been characterized at diverse resolution scales. The DNA is composed by nucleotides forming base pairs ([A], an AT base-pair from PDB entry 2KV0 [48]), which in turn will form a DNA double helix ([B], DNA structure from PDB entry 2KV0 [48]). The DNA then wraps around histone proteins forming nucleosomes ([C], the complex between nucleosome core particles and DNA from PDB entry 1AOI [49]). It is also known that chromosomes occupy so-called chromosome territories ([F], 3D FISH image from a 3D map of all chromosomes in human male fibroblast nuclei [50]). Between DNA atomic resolution and nuclei chromosome resolution, there have been a plethora of models describing how chromatin folds into the so-called 30 nanometer fiber ([D], image by Richard Wheeler) and then experiences higher-order folding ([E], interchromatin domain and interchromosomal network models of looping interactions between two chromosomes [51]). An integrative approach combining polymer physics with constraint-based modeling will provide important insight about chromatin architecture at the range of resolutions indicated by the dashed rectangle. Length, volume, and resolution scales adapted from [52].

We present two complementary approaches to address this challenge: (i) the first approach aims at developing simple polymer models of chromatin and determining relevant interactions (both physical and biological) that explain experimental observations; (ii) the second approach aims at integrating diverse experimental observations into a system of spatial restraints to be satisfied, thereby constraining possible structural models of the chromatin. The goal of both approaches is dual: to obtain most accurate 3D and 4D representation of chromatin architecture and to understand physical constraints and biological phenomena that determine its organization. These approaches are reminiscent of the protein-folding field where the first strategy was used for characterizing protein “foldability” and the second was implemented for modeling the structure of proteins using nuclear magnetic resonance and other experimental constraints. In fact, our outlook consistently returns to the many connections between the two fields.

## What Does Technology Show Us?

**Figure 2:**
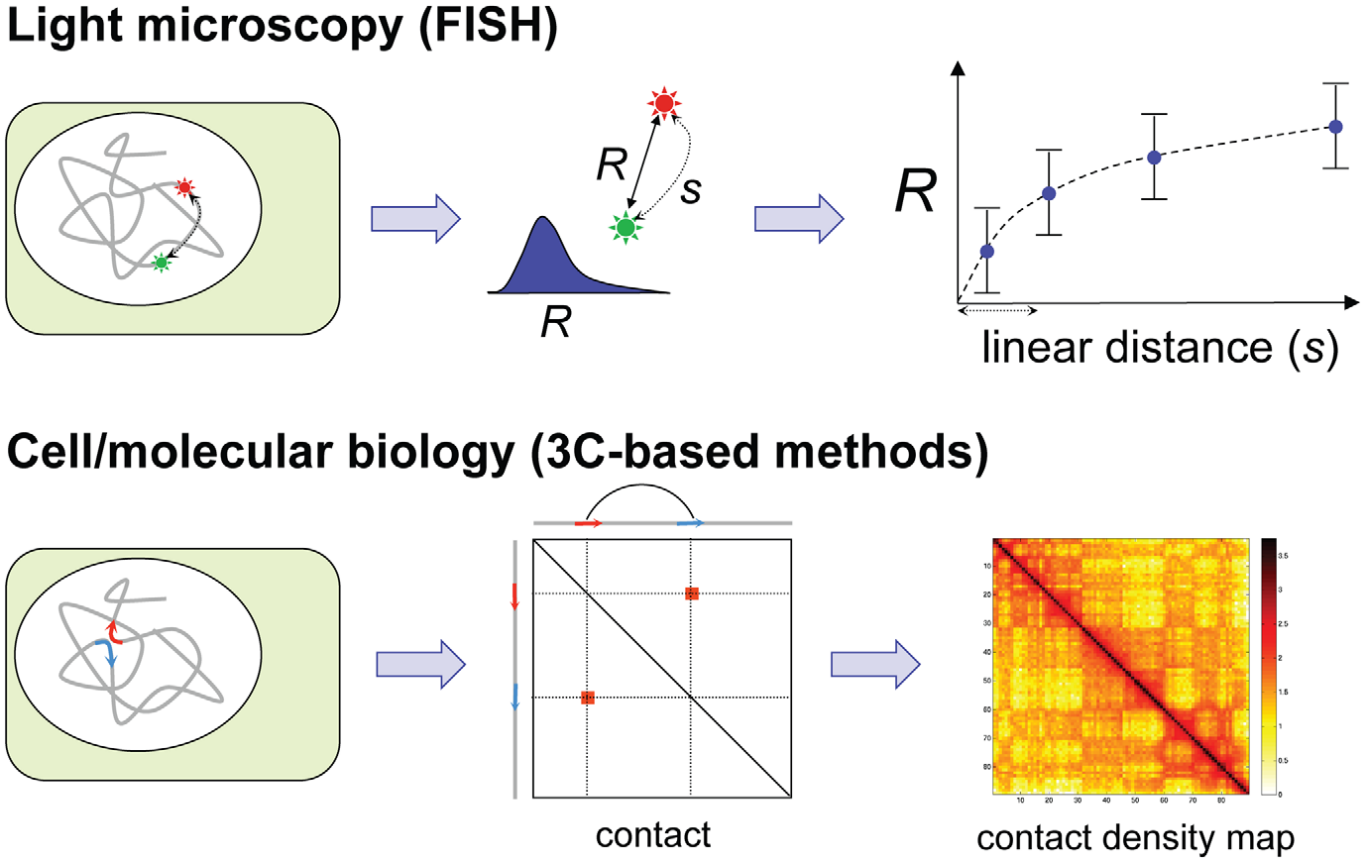
Main approaches for studying genomic organization. Two of the most used approaches for experimentally determining features of genome architecture. Light microscopy by fluorescent *in situ* hybridization (FISH) results in a measured spatial distance (*R*) (and its distribution in a population of cells or its time course) as function of the genomic linear distance (*s*). Cell/molecular biology by chromosome conformation capture (3C)-based approaches results in an estimation of the average frequency of contacts between parts of the chromatin in a population of cells.

Today, it is possible to quantitatively study structural features of genomes at diverse scales that range from a few specific loci, through chromosomes, to entire genomes (Table 1) [3]. Broadly, there are two main approaches for studying genomic organization: light microscopy and cell/molecular biology (Figure 2). Light microcopy [4], both with fixed and living cells, can provide images of a few loci within individual cells [5], [6], as well as their dynamics as a function of time [7] and cell state [8]. On a larger scale, light microscopy combined with whole-chromosome staining reveals chromosomal territories during interphase and their reorganization upon cell division. Immunofluorescence with fluorescent antibodies in combination with RNA, and DNA fluorescence *in situ* hybridization (FISH) has been used to determine the co-localization of loci and nuclear substructures.

**Table 1:** Experimental genome structure analysis.

Method	Type	Scale	Output	Reference
RNA FISH	Single cell	Genome-wide	Images	[4]
DNA FISH	Single cell	Genome-wide	Images	[4]
High-res. FISH	Single cell	Genome-wide to intermediate (Mb)	Images	[4]
DamID	Population	Genome-wide	DNA-lamina interactions	[14]
Hi-C	Population	Genome-wide	Chromatin fiber interactions	[13]
4C	Population	Genome-wide to intermediate (Mb)	Chromatin fiber interactions	[10], [11]
5C	Population	Intermediate (Mb)	Chromatin fiber interactions	[12]
3C	Population	Fine (Kb)	Chromatin fiber interactions	[9]

Kb, kilobases; Mb, megabases. Table adapted from [3], [47].

Using cellular and molecular biology, novel chromosome conformation capture (3C)-based methods such 3C [9], 3C-on-chip or circular 3C (the so-called 4C) [10], [11], 3C carbon copy (5C) [12], and Hi-C [13] quantitatively measure frequencies of spatial contacts between genomic loci averaged over a large population of fixed cells. 3C-based approaches have been applied to individual genomic regions and entire genomes, and provide data with resolution ranging from tens of kilobases (Kb) to megabases (Mb). Mapping interactions between genomic regions and the nuclear lamina provides additional information about genomic spatial organization [14]. Finally, measuring the responses to physical forces characterizes mechanical properties of chromosomes [15].

The listed experimental approaches are largely complementary in their advantages and limitations. While light microscopy can only characterize a limited number of loci in a small number of cells, its single-cell resolution makes it a preferred technology for characterizing chromatin variability and dynamics [16]. Conversely, while 3C-based approaches provide high-resolution contact frequencies for large genomic domains or entire genomes, they do not provide information about individual cells. Instead, 3C measurements report ensemble-averaged properties of genomic conformations in a large population of cells (typically more than a million cells).

All of these techniques have helped to characterize intriguing features of genome organization during interphase. We now know that in human cells chromosomes occupy distinct *chromosomal territories* [17] and are organized into alternating active and inactive chromatin domains with many long-range interactions [13]. Most importantly, these experimental techniques have demonstrated that chromosomes adopt highly dynamic conformations related to the functional state of their genes. The development of biophysical models of higher-order chromatin architecture based on these new data helps to elucidate the organizing principles of genomes and constitutes, by itself, an emerging field of computational biology.

## What Does Physics Tell Us?

Application of polymer physics to protein folding led to major breakthroughs in understanding the mechanisms of folding [18], [19] and design principles of natural foldable proteins [18], [20]. Statistical mechanics of polymers has also been successfully applied to characterize physical properties of DNA (e.g., [21]–[26]), but less so to chromatin fibers and their organization into interphase and metaphase chromosomes [13], [27]–[29]. The availability of rich new imaging and 3C-based data is clearly changing this trend.

**Figure 3:**
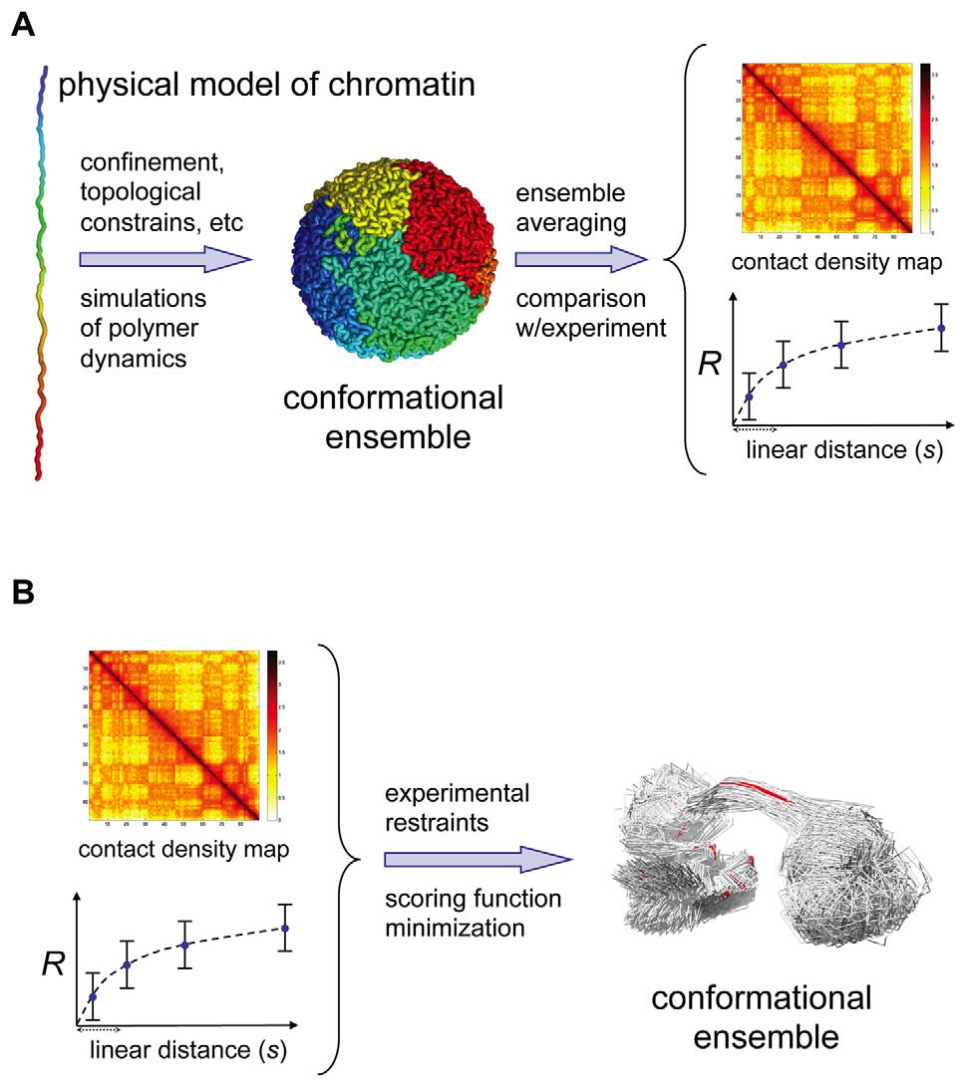
Two computational approaches for determining the 3D structure of genomic domains and genomes. (A) The first approach uses polymer models to simulate relevant interactions (both physical and biological) that explain experimental observations. (B) The second approach integrates diverse experimental observations to model a conformational ensemble that satisfies the experimental observations.

In contrast to the majority of proteins that fold into unique native conformations, a chromatin fiber is likely to have different conformations in individual cells, forming an ensemble of conformations. It remains to be seen how diverse this ensemble is and, by analogy to protein folding, whether it resembles an unfolded state of a protein or a transition state ensemble. One drastic difference between proteins and chromatin is the length of the polymer. While single protein domains have a ratio of length to chain diameter of ∼50–250 (that is, 50–250 amino acids), yeast chromosomes yield the ratio of ∼10^3^–10^4^ (that is, 200–1,500 Kb, 10 nm fiber diameter, 7 fold packing by nucleosomes) and ∼10^5^–10^6^ for human chromosomes (that is, 50–250 Mb). These extraordinary long polymers cannot be organized into structures as ordered as that of proteins, and presumably remain largely disordered. The goal of the computational approach is to determine what sort of polymer models and interactions can generate conformational ensembles that are consistent with experimental data (Figure 3A). Experimental features that can be used to test the model include contact probability obtained by 3C-based experiments, the distribution of the spatial separation as a function of genomic distance between two loci [29], formation of domains of active and inactive chromatin, existence of chromosomal territories, etc. One can also seek models that reproduce experimentally observed dynamics of chromosomal loci (e.g., displacement of a locus as a function of time [7] or upon gene activation [8], [30]). Finding an appropriate model involves representing chromatin as a polymer and simulating its dynamics subject of physical interactions (e.g., spatial and topological constraints, confinement, and supercoiling) as well as biological interactions (e.g., specific and non-specific interactions between chromosomal loci, and nuclear lamina/matrix, among others).

Recent studies provide many examples of successful use of polymer physics in describing chromosome architecture. A recent study of the human chromatin using the Hi-C technique has shown that statistics of long-range interactions are consistent with a long-lived non-equilibrium state of a homopolymer emerging due to rapid condensation, rather than with any particular equilibrium state [13]. Approaching this problem using polymer physics can also reveal the roles of excluded volume, chain entropy, confinement, DNA supercoiling, and topological constraints in shaping the conformational ensemble of chromatin. For example, recent studies of short polymer rings suggested that topological constraints may be sufficient for the maintenance of chromosomal territories in eukaryotes [31], [32]. Similarly, the entropy of the DNA chain was suggested to be sufficient for segregation of chromosomes during *E. coli* division [33]. A final example is that a quasi-linear organization of the circular *E. coli* chromosome was shown to be consistent with a model where DNA supercoiling plays a central role [5]. Since several alternative physical models may fit even the most data-rich experiments equally well, follow-up experiments are required to dissect alternative models.

## What Can We Learn from Data Integration?

Data integration using computational approaches has already proven useful in the determination of structures of large complexes of proteins. In a landmark study addressing this problem, the Sali Lab (University of California San Francisco) used the Integrative Modeling Platform (IMP, http://www.integrativemodeling.org/), a multi-scale and flexible computational framework based on the satisfaction of spatial restraints [34]. In IMP, the problem of determining a probabilistic map of all proteins in the nuclear pore complex (NPC) was expressed as an optimization problem, where all available experimental information was integrated and represented as spatial restraints. The systematic integration of the input information provided a more complete and detailed structure of the NPC than any of the independent experimental observations could reach [35].

Similar integration of results using computational approaches are now being successfully applied towards the structural determination of genomic domains and genomes (Figure 3B) [36]. For example, the use of light imaging by FISH and computation resulted in a low-resolution architecture of the immunoglobulin heavy-chain (*Igh*) locus [37]. By using a set of 12 fluorescent probes spanning the entire *Igh* locus, Murre and co-workers proposed that the *Igh* locus is organized into compartments containing clusters of loops separated by linkers. The integration of higher resolution experimental techniques such as 3C-based methods with computation has also resulted in high resolution-models of the HoxA cluster [38], two fungi genomes (*S. cerevisiae* [39] and *S. pombe* [40]), and the human α-globin domain [41]. The availability for the first time of high-resolution 3D models of genomic domains and genomes confirms and expands our knowledge of the higher-order folding of chromosomes. For example, the analysis of the 3D models of the human α-globin domain [41] have shown that long-range interactions between active functional elements are sufficient to drive folding of local chromatin domains into compact globular states [42]. Finally, data integration can also be used to examine genomes in 4D by incorporating dynamics into an objective function.

## Future Outlook

The conceptual framework outlined here allows the integration of data from different experimental sources with a proper treatment of chromatin physics. However, this approach will face several challenges, such as: (i) identifying the proper *representation* of the chromatin that matches the resolution of the diverse experimental observations, (ii) correctly translating the experimental observations into the modeled properties of chromatin and objective function(s) that can be minimized (*scoring*), and (iii) finding a balance between the level of representation that captures the essential physics, while allowing an adequate search of the conformational space (*sampling*) using available computational resources. Nevertheless, we trust that the approaches outlined here for determining the spatial organization of chromatin may prove very useful not only for identifying long-range relationships between genes and distant regulatory elements, but also for elucidating chromatin higher-order folding principles. Such technology will indeed contribute to the characterization of the relationship between sequence, structure, and function for entire genomes.

We foresee that reconstructing conformational ensembles of genomic domains and genomes via the integration of experimental results with computational analysis will help answer many fundamental questions. For example, if the molecular rules of chromosome organization involve DNA, proteins, and other nucleic acids, how can local interactions between these building blocks, which are three orders of magnitude smaller than the size of a chromosome, determine chromosome organization, re-organization, cell-to-cell variability, and dynamics [43]? How does chromatin architecture constrain or facilitate a range of biological processes that require direct access to the genetic information (i.e., to the naked DNA) [44]? How does chromatin, which constitutes a significant fraction of the nuclear volume, limit diffusive mobility of other proteins and nucleic acids within the nucleus [45]? What is the role of higher-order chromatin architecture in coordinating expression of several proximal or distant genes, allele-specific expression, and activation and inactivation of genomic loci and whole chromosomes [46]? Chromatin organization can also be influenced by large-scale characteristics of an organism, including genome length, the number of chromosomes, ploidy, nuclear shape and volume, and location and anchoring of centromeres and telomeres. Modeling can shed light on how these factors shape chromosomes in different organisms, at different stages of cell life, and the occurrence of chromosomal aberrations in cancer.

Proper integration of experimental results and their interpretation in light of polymer physics can only result in improved models of how chromosomes fold in the interphase nucleus. With the increasing accuracy and flexibility of integrative approaches, we envision a wide spread of applications. The participation of the structural computational biology community will be crucial for curating, organizing, and disseminating the wealth of incipient data. We invite readers to participate in open discussions of these questions and approaches by visiting http://www.3dgenomes.org/.

**Author Biographies**

**Marc A. Marti-Renom** has a PhD in biophysics from the Universitat Autònoma de Barcelona where he worked on protein folding under the supervision of Professors B. Oliva, F. X. Avilés, and M. Karplus. After that, he went to the United States for his postdoctoral training on protein structure modeling at the Sali Lab as the recipient of the Burroughs Wellcome Fund fellowship at the Rockefeller University. Later on, he was appointed Assistant Adjunct Professor at the University of California San Francisco (UCSF). Since 2006, he has been head of the Structural Genomics Laboratory (http://sgu.bioinfo.cipf.es/) at the Centro de Investigación Príncipe Felipe (CIPF) in Spain. His group is broadly interested in how RNA, proteins, and genomes organize and regulate cell fate. Dr. Marti-Renom is an Associate Editor with *PLoS Computational Biology*.

**Leonid A. Mirny** gained a PhD in biophysics from the laboratory of Eugene Shakhnovich at Harvard University where he worked on several problems in protein folding and evolution. After serving as a Junior Fellow at Harvard Society of Fellows, he joined the faculty of the Harvard-MIT Division of Health Science and Technology and the Department of Physics at MIT. The Mirny Lab has been working on a range of problems in biophysics, including analysis of biological networks, mechanism of protein-DNA search and cooperative binding, and higher-order chromatin organization.
